# arriba-lib: evaluation of an electronic library of decision aids in primary care physicians

**DOI:** 10.1186/1472-6947-12-48

**Published:** 2012-06-06

**Authors:** Oliver Hirsch, Heidemarie Keller, Tanja Krones, Norbert Donner-Banzhoff

**Affiliations:** 1 Department of General Practice/Family Medicine, University of Marburg, Marburg, Germany

## Abstract

**Background:**

The successful implementation of decision aids in clinical practice initially depends on how clinicians perceive them. Relatively little is known about the acceptance of decision aids by physicians and factors influencing the implementation of decision aids from their point of view. Our electronic library of decision aids (arriba-lib) is to be used within the encounter and has a modular structure containing evidence-based decision aids for the following topics: cardiovascular prevention, atrial fibrillation, coronary heart disease, oral antidiabetics, conventional and intensified insulin therapy, and unipolar depression. The aim of our study was to evaluate the acceptance of arriba-lib in primary care physicians.

**Methods:**

We conducted an evaluation study in which 29 primary care physicians included 192 patients. The physician questionnaire contained information on which module was used, how extensive steps of the shared decision making process were discussed, who made the decision, and a subjective appraisal of consultation length. We used generalised estimation equations to measure associations within patient variables and traditional crosstab analyses.

**Results:**

Only a minority of consultations (8.9%) was considered to be unacceptably extended. In 90.6% of consultations, physicians said that a decision could be made. A shared decision was perceived by physicians in 57.1% of consultations. Physicians said that a decision was more likely to be made when therapeutic options were discussed “detailed”. Prior experience with decision aids was not a critical variable for implementation within our sample of primary care physicians.

**Conclusions:**

Our study showed that it might be feasible to apply our electronic library of decision aids (arriba-lib) in the primary care context. Evidence-based decision aids offer support for physicians in the management of medical information. Future studies should monitor the long-term adoption of arriba-lib in primary care physicians.

## Background

Decision aids are designed to help patients make informed choices among diagnostic or treatment options by delivering evidence-based information on options and outcomes. They should supplement the counselling process and can be delivered in different formats before, during or after the consultation [1]. Most of them are designed to be viewed by the patient prior to the consultation. They can be educational, prescriptive (promoting a certain decision), or descriptive (promoting the process of deliberation). They are reported to increase knowledge, reduce decisional conflict, cause greater satisfaction with decision making, support more realistic expectations, achieve a greater likelihood of being able to make a decision, result in an increased association between patient values and decisions, support patient participation, and enhance communication between physicians, patients and their relatives [2]. Decision aids should not substitute personal counselling because uncertain patients would then be unable to have direct discussions with medical experts in order to make a sound decision [3].

Several authors argue for the need to develop evidence-based decision aids for a wide range of clinical applications. They should display this evidence on a basic level to be understandable to the patient. Decision aids could also be interactive so that individual risk data can be entered and the effects of certain treatments can immediately be seen. Potential sources of error (e.g. inaccurate data entry, comprehension errors) should be kept to a minimum. Pros and cons can, for example, be discussed by using weigh scales to ensure the incorporation of patients´ values [4]. The successful implementation of decision aids in clinical practice depends on how clinicians perceive them [5-7]. In her survey among an interdisciplinary group of clinicians, Ruland identified several important features for the implementation of decision support systems in clinical practice. They should be easy to use without increasing workload, and should deliver updated, precise information [8]. On the patient side, the achievement of congruence between patient preferences and expectations was also regarded as an important factor. There was a high acceptance regarding the usefulness of evidence- and preference-based decision aids among clinicians, especially in the outpatient setting. Ruland concludes that more attention should be directed to the development and implementation of such tools. Graham et al. state that relatively little is known about the acceptance of decision aids by physicians and the factors influencing the implementation of decision aids from their point of view [9]. In their study, a large majority appreciated the quality of the decision aids and valued their usefulness for patients. Nevertheless, there was a wide gap between intention to use decision aids in the future and the actual use. For example, only half of the family physicians who indicated that they would use the presented decision aid on hormone replacement therapy actually used it in their clinical practice. Logistical matters, like expected time constraints, seemed to most strongly influence the implementation in the respective practices. The aforementioned results were also confirmed by Thistlethwaite et al. [10]. Physicians mentioned that they need more training in shared decision making and in the use of decision aids. There was a tendency to use decision aids as educational resources rather than as interactive tools. This also points to the need to develop user-friendly decision aids that can be used within consultations.

Decision aids have rarely been field tested to assess patients’ and physicians’ attitudes towards them [11], although recently more attempts to do so were made [12-15]. Further, there is a lack of evidence showing how decision aids are implemented in clinical practice [16]. It is therefore strongly recommended to evaluate decision support systems in a real world setting with multi-perspective, multi-method studies before they are disseminated for routine use. Such studies should contain a variety of aspects, use multiple methods, apply flexible study designs with longitudinal measures, and perform formative and summative evaluations. Most studies in this area involve only physicians, not patients or other users [4,11,13,17,18].

The aim of our study was to evaluate the uptake of an interactive, transactional, and evidence-based library of decision aids and its association to decision making in patients and physicians in the primary care context. We undertook a mixed methods evaluation study using quantitative and qualitative methods. Here, we report quantitative results relevant for the evaluation and implementation of a library of decision aids in primary care physicians. Detailed analyses of our qualitative material will be published in additional papers. Based on the new Medical Research Council guidance on complex interventions, our study can be regarded as an evaluation study [19]. Such studies can provide important information to stimulate and direct research in specific areas [20].

## Methods

### arriba-lib

Our electronic library of decision aids, arriba-lib, is an extension of ARRIBA-Herz, a decision aid on cardiovascular prevention that was investigated in a randomised controlled trial [21] and which is now named “arriba™”. The software, where “lib” is an acronym for “library”, has a modular structure and presently contains evidence-based decision aids for several topics: cardiovascular prevention, atrial fibrillation, coronary heart disease, oral antidiabetics, conventional and intensified insulin therapy, and unipolar depression. Further modules are being developed.

**Figure 1:**
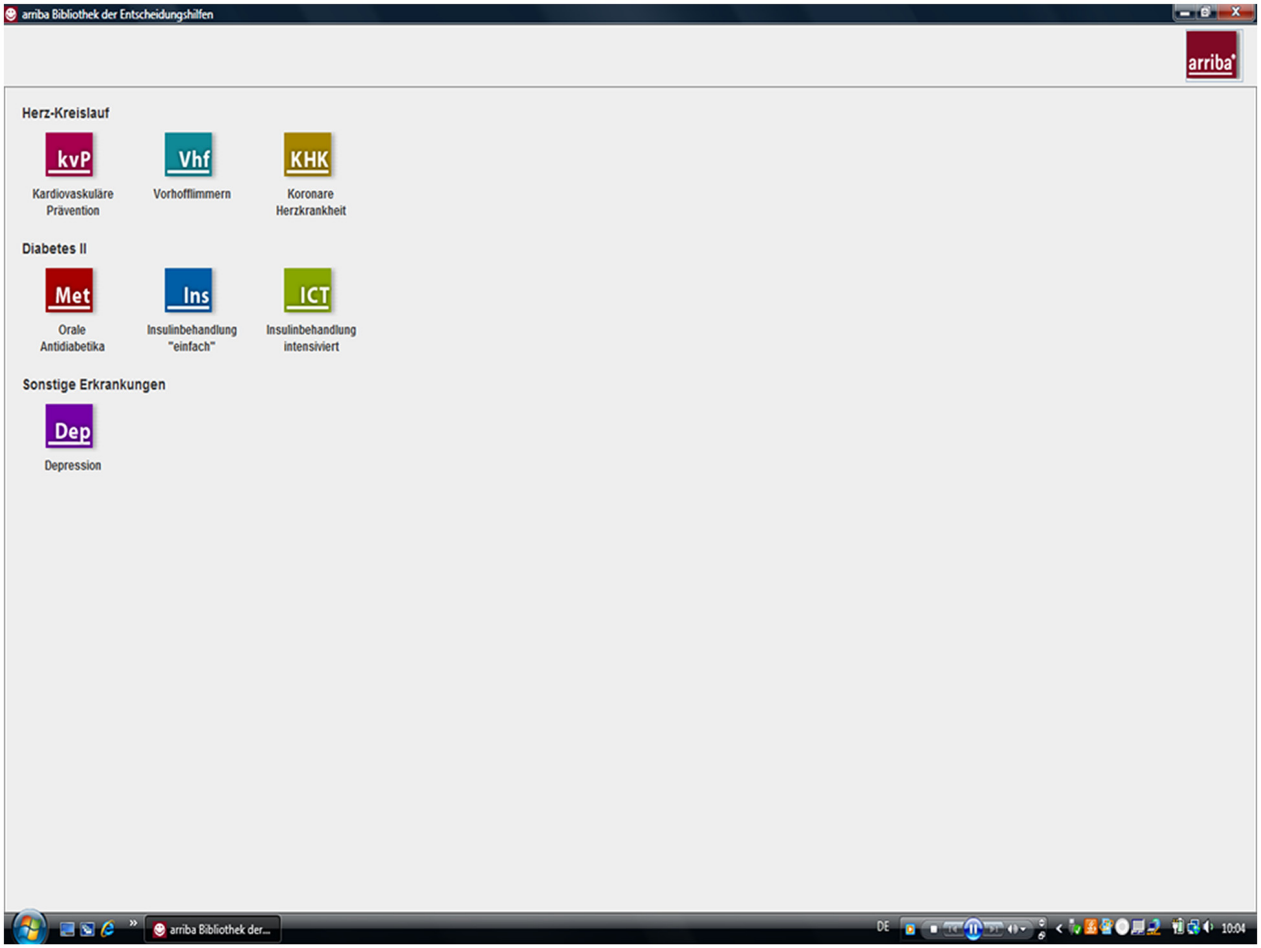
Opening screen of arriba-lib.

Figure 1 displays the opening screen of arriba-lib and shows the library-like structure.

Arriba-lib is a java application that does not require an installation process and is less than 15 megabytes in size.

**Figure 2:**
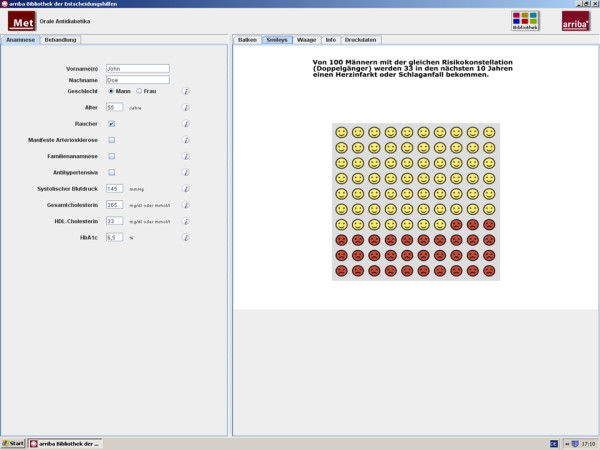
Individual risk information with emoticons.

The modules are structured to assist physicians in counselling their patients according to the philosophy of shared decision making [22,23]. Our programme includes the following successive steps: (1) definition of the problem, (2) discussion of the individual risk, (3) discussion of treatment options, (4) deliberation, and (5) plan for future actions. These steps can be regarded as a framework to help the clinician effectively structure the encounter. After entering history information, individual risk information is displayed by emoticons, bar charts, or curves. For example, within the module regarding oral antidiabetics (metformin), Figure 2 shows the use of emoticons to illustrate the risk of suffering from a myocardial infarction or stroke in the next ten years compared to one hundred patients with the same characteristics. These emoticons are an easily understandable graphic representation of global risk information which considers the limited numeracy and statistical literacy of patients and physicians [24,25]. The presentation of risk information was shown to increase the accuracy of perceived risk [26].

**Figure 3:**
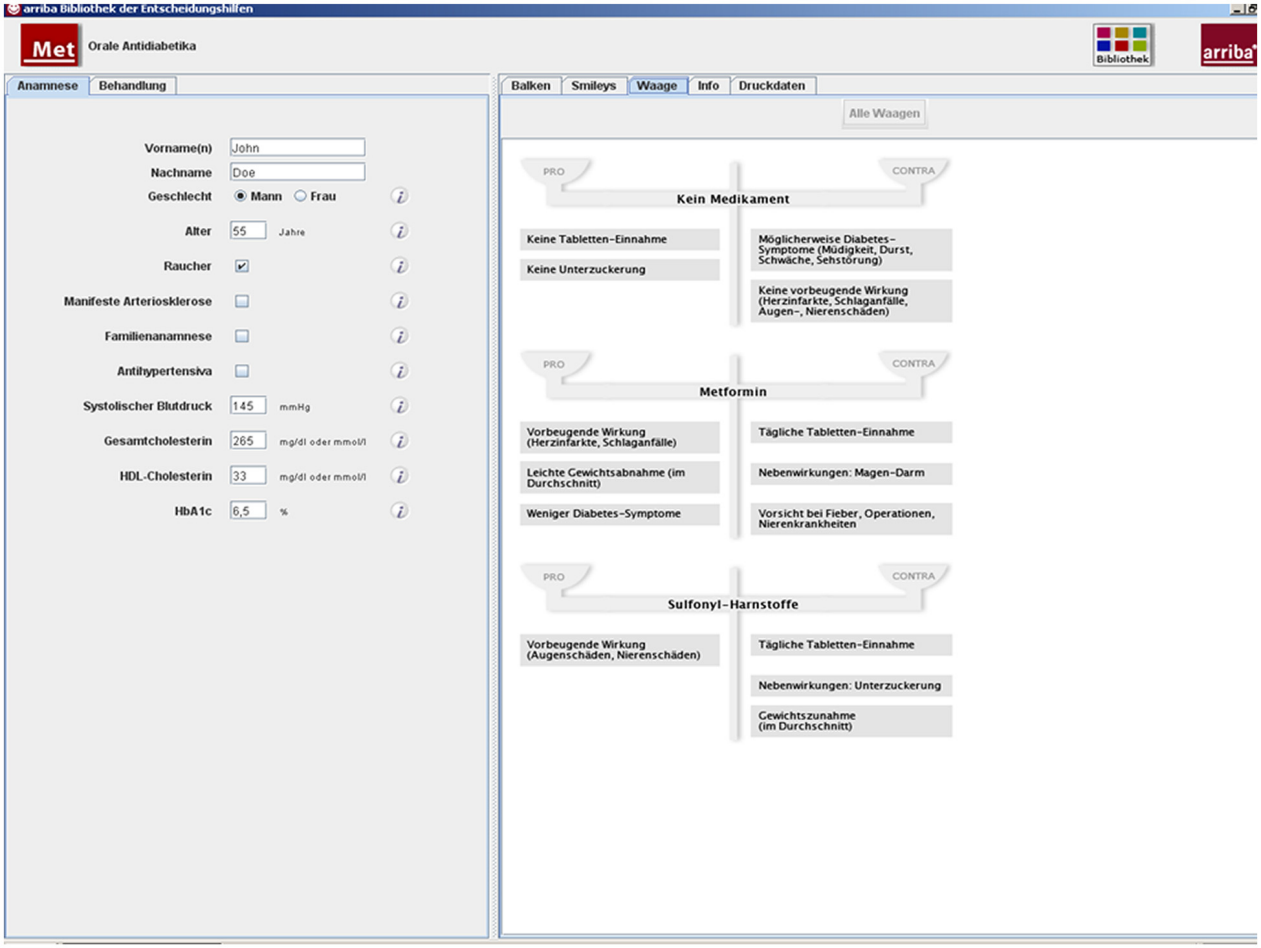
Weigh scales in arriba-lib for the deliberation phase.

After having chosen between evidence-based treatment options, risk-reducing effects can be demonstrated by the changed appearance of emoticons. The process of deliberation can be supported by weigh scales mentioning pros and cons related to each option (Figure 3).

Additional evidence-based information on covered topics and on communication strategies is also provided in the programme and can be easily accessed in each module. For the purpose of our study, log files of every consultation were created that recorded every step taken in the modules and how long it took to initiate the next step.

The participating physicians received a personal introduction into the programme and the philosophy of shared decision making in several formats. Members of our research team visited the participating physicians, gave an introduction to the modules of our electronic library, and conducted an interactive seminar on shared decision making. Training lasted about 90 minutes. Physicians also received written information about our electronic library and about shared decision making via a 50 page booklet. This training approach and the provided material were evaluated positively by the participating physicians.

### Recruitment and sampling

We asked a convenience sample of 91 primary care physicians in the German federal lands of North Rhine-Westphalia and Hesse to participate in our study, of whom 34 agreed. Five of these 34 physicians failed to recruit patients so that 29 primary care physicians included 192 patients. Patients were included when there was a decision to be made in the topics covered by arriba-lib. Forty-five patients were excluded from participation because 27 refused to participate and physicians regarded 18 patients as not being able to participate (restrictions because of language, cognitive abilities, psychiatric disorder, and severity of somatic disease). On average, recruitment of patients comprised a period of eight weeks.

**Figure 4:**
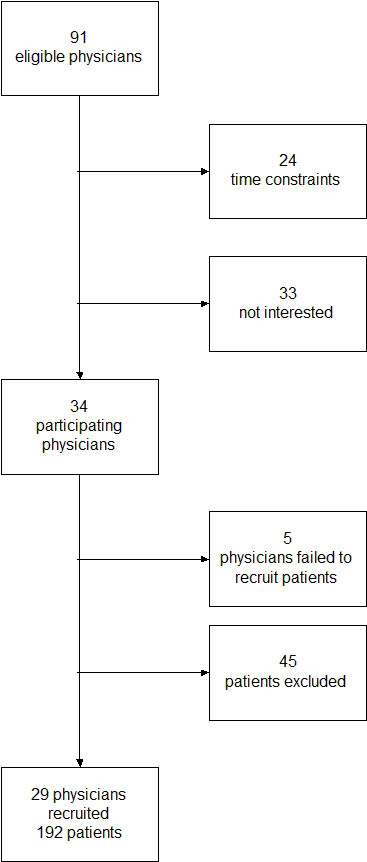
Flow chart displaying the recruitment process in the arriba-lib study.

The recruitment process is depicted in Figure 4.

The study complies with the Declaration of Helsinki. The research protocol was approved by the local research ethics committee at the University of Marburg in Germany. All physicians and patients gave their informed consent.

### Measurements

The physician questionnaire contained information on which module was used and how detailed the steps of the shared decision making process (definition of the problem, discussion of the individual risk, discussion of treatment options, deliberation, and plan for future actions) were discussed using a four point scale (“not at all”, “hardly”, “detailed”, “very detailed”). We further asked physicians who made the decision at the end of the consultation, and they gave a subjective appraisal of consultation length (“unacceptably extended”, “acceptably extended”, “neither nor”, “shortened”).

### Data analyses

Because of the hierarchical structure of our data (patients nested within physicians), we used generalised estimation equations [27] to measure associations between patient and physician variables [28]. The Wald χ^2^ test was used as a test statistic. To enhance the interpretability of the results, we also analysed the data with traditional crosstab analyses (χ^2^ test, Haldane-Dawson Test). The effect size Cramer V (.30 and higher denotes a strong effect) was used to measure the meaning of associations [29]. As we did 10 different analyses, the adjusted significance level would be α=.05/10 = .005 according to the Bonferroni method. This must be considered when interpreting the results [30].

After inspectioning descriptive data, there was a maximum of 10% missing data on isolated variables which can be classified as missing completely at random because there were no patterns of associations with other variables [31]. Imputation of missing data was done by inserting the means of the respective variables on the physician level, which in simulation studies was found to be most appropriate when the data has a hierarchical structure [32].

## Results

The average age of the participating primary care physicians was 52.2 years (sd 5.1 years; range: 43 to 64 years). Eighteen were male (62%) and eleven were female (38%). The average time practising was 14 years (sd 7.5 years).

The module for cardiovascular prevention was selected in 128 patients (67%), the diabetes modules in 43 patients (22%), coronary heart disease in 8 patients (4%), atrial fibrillation in 8 patients (4%), and depression in 3 patients (2%).

The medium age of the 192 participating patients was 62.4 years (sd 11.8 years). There was an equal distribution regarding gender with 97 males (50.5%) and 95 females (49.5%). A majority of 70.3% had a formal education of 8 years or less, 14.6% had a formal education of up to 10 years, and 15.1% of more than 10 years. In our sample, 122 patients (63.5%) preferred shared decision making with their physicians, and exactly the same number mentioned that shared decision making actually had taken place.

In 46 patients (24.0%) the reason for consultation was a check-up, 34 patients (17.7%) attended their physicians for a follow-up visit regarding a previously presented problem, and 20 patients (10.4%) were seen in the context of a disease management programme. The remaining patients came with an acute medical problem or to discuss results of laboratory examinations.

Physicians stated that in 30.9% of consultations alternative therapeutic options had been discussed for the first time, in 49.7% of consultations options had been discussed before, in 17.3% previous therapeutic measures were re-evaluated, and in 2.1% more than one of these options was selected.

In 136 patients (70.8%) a special appointment was made to discuss the clinical problem.

Regarding the subjective appraisal of consultation length, in 8.9% of consultations physicians said that they were “unacceptably extended” by the use of arriba-lib, 76.3% of consultations were “acceptably extended”, 14.2% “neither nor”, and 0.5% were “shortened”.

### Steps of the shared decision making process in arriba-lib

Physicians´ ratings on how detailed the steps of the shared decision making (SDM) process were discussed during the consultations are depicted in Table 1.

**Table 1:** Physicians ratings on how detailed the steps of the shared decision making process were discussed during the consultations (n = 192)

**SDM step**	**Very detailed**	**Detailed**	**Hardly**	**Not at all**
definition of the problem	13 (7.3%)	62 (32.3%)	96 (50.0%)	20 (10.4%)
discussion of the individual risk	37 (19.3%)	136 (70.8%)	19 (9.9%)	0 (0.0%)
discussion of treatment options	36 (18.8%)	125 (65.1%)	31 (16.1%)	0 (0.0%)
deliberation	27 (14.1%)	119 (62.0%)	46 (23.9%)	0 (0.0%)
plan for future actions	16 (8.3%)	120 (62.5%)	55 (28.7%)	1 (0.5%)

The data in Table 1 reveals that the definition of the problem was discussed in less detail compared to the other steps and that the individual risk and treatment options were discussed most thoroughly.

The subjective duration of consultations was independent from how detailed the steps of the SDM process were discussed. As an example, Table 2 shows the non-significant association between subjective duration of consultations and the detailedness of discussion of individual risk (Haldane-Dawson Test: z = 0.65, p = .52; Cramer V = .15).

**Table 2:** Cross tabulation of the detailedness of discussion of individual risk and the subjective duration of consultations

	**Discussion of risk**			
**Duration**	**Not at all**	**Hardly**	**Detailed**	**Very detailed**
shortened	0 (0%)	0 (0%)	1 (100%)	0 (0%)
neither nor	0 (0%)	5 (18.5%)	21 (77.8%)	1 (3.7%)
acceptably extended	0 (0%)	14 (9.7%)	99 (68.3%)	32 (22.0%)
unacceptably extended	0 (0%)	0 (0%)	13 (76.5%)	4 (23.5%)

### Association of arriba-lib with decision making

In 90.6% of consultations, physicians said that a decision could be made. The decision was perceived as shared by physicians in 57.1% of consultations, the physicians thought that they had decided on their own in 32.4% of consultations, while they stated that their patients mainly made the decision in 10.6% of consultations.

Physicians perceived that a decision was more likely to be made when therapeutic options were discussed “detailed”, and that a decision was less likely when therapeutic options were discussed “very detailed” (GEE: Wald-χ^2^ =6.72, p = .01). A limiting factor regarding this interpretation is the relatively small number of consultations in which no decision could be made (Table 3).

**Table 3:** Cross tabulation of the detailedness of discussion of options and physicians´ indications that a decision could be made

	**Decision**	
**Discussion of options**	**Yes**	**No**
not at all	0 (0.0%)	0 (0.0%)
hardly	29 (16.7%)	2 (11.1%)
detailed	115 (66.1%)	10 (55.6%)
very detailed	30 (17.2%)	6 (33.3%)
total	174 (100%)	18 (100%)

No significant associations were found between the physician´s indication that a decision could have been made and the subjective duration of the consultation or the reason for the consultation. Additionally, we found no significant associations between the physician´s appraisal of who had made the decision and the detailedness of the discussion on the SDM steps.

### Prior experience with decision aids

Prior experience with the precursor of arriba-lib was not significantly associated with the detailedness of the discussion of the SDM steps (P values in the GEE model ranged from .19 to .58 for the single steps). There was also no significant association between this prior experience and the selection of the different arriba-lib modules (χ^2^ =8.68, p = .12). We also found no meaningful associations between prior experience with a decision aid and the subjective duration of consultations (GEE: Wald-χ^2^ =1.17, p = .28), or physicians´ indications that a decision could be made (GEE: Wald-χ^2^ =2.92, p = .09), or physicians´ indications of who had made the decision (χ^2^ =3.66, p = .30).

## Discussion

We conducted an evaluation study of an interactive, transactional, and evidence-based library of decision aids in primary care physicians and examined the underlying steps of the SDM process, and the influence of prior experience with decision aids.

The subjective duration of consultations was not significantly associated with the subjective appraisal of how detailed the steps of the SDM process were discussed. In 8.9% of consultations physicians said they were unacceptably extended and in 90.6% of consultations, physicians said that a decision could be made. A shared decision was perceived by physicians in 57.1% of consultations. Physicians perceived that a decision was more likely to be made when therapeutic options were discussed “detailed” and that a decision was less likely when therapeutic options were discussed “very detailed”.

Prior experience with the precursor of arriba-lib was not a critical variable within our sample of primary care physicians. We did not find significant associations between prior experience and detailedness of the discussion of the SDM steps, selection of arriba-lib modules, subjective duration of consultations, physicians´ indications that a decision could be made, or physicians´ indications of who had made the decision.

Our study has several limitations. In our evaluation study we had no control group so that we cannot compare our results to the situation of usual care. It is likely that the participating physicians did not consistently perform consecutive patient recruitment. This might have led to a positive selection of patients who were already favourably disposed to SDM. This positive selection bias concerning SDM might also hold true for the participating physicians because just 32% of eligible physicians took part in the study. The wording of our four point scale (not at all”, “hardly”, “detailed”, “very detailed”) might have been problematic. The most common choice might be located between “hardly” and “detailed”. Results of statistical analyses within an evaluation study should always be treated with caution and should be regarded as preliminary [30]. As we did 10 different analyses, the adjusted significance level would be α=.05/10 = .005 according to the Bonferroni method. The α level of our only significant result (p = .01) slightly exceeded this level after correcting for multiple testing.

There is a limited database in Germany showing how physicians perceive the concept of SDM and whether they have the necessary basic communicative skills for SDM [33]. A telephone survey of 502 physicians and 1512 German citizens revealed that 67% of physicians preferred a shared decision. There were no differences regarding gender or speciality, but younger physicians were more likely to favour SDM. In their nationally representative sample of U.S. physicians, Murray et al. found that three quarters preferred shared decision making with their patients [34]. In our sample, physicians say in about 75% of consultations based on shared decision making that they were extended in an acceptable time frame. This finding is also corroborated by Nannenga et al. [35]. There seems to be a threshold in physicians´ perceptions when a decision can be made. This is supported by our finding that, according to our physicians, a decision is more likely when therapeutic options are discussed “detailed” and it is less likely when therapeutic options are discussed “very detailed”. A very detailed discussion of therapeutic options might lead to an information overload in patients and therefore exceed a threshold of indecision. It may further indicate that physicians and/or patients feel uncertain and need more time to discuss possible options which may not necessarily result in a decision. The analysis of log files, which is presented in detail in another publication [36], showed that the average consultation time was 8 minutes. In Germany, primary care physicians are mainly paid for patient contacts of 10 minutes. Therefore, the use of decision aids did not extend the average consultation time. We found discrepancies between these subjective appraisals of the detailedness of shared decision making steps and the log data, which represents user interactions with our electronic library of decision aids. It was possible to record the time that was spent with a certain option within the modules (e.g. emoticons) and we were therefore able to calculate the proportion of consultation time spent with specific features. In the cardiovascular prevention module, 35 of 122 consultations (28.7%) spent 100% of consultation time in the history part of the programme, which includes risk presentation. These consultations were shorter than average. In the other modules with weigh scales, 15 of 62 consultations (24.2%) spent 100% of consultation time in the history part; 11 of these consultations used the oral antidiabetics module. Again, these consultations were shorter than average. In contrast, all of the physicians indicated in their subjective appraisals of the detailedness of shared decision making steps that therapeutic options were discussed. In these consultations, physicians obviously discussed therapeutic options with their patients without using the respective modules which points to a reduced fidelity in this point [5]. They might have preferred different ways of discussing them, or they did not agree with the evidence-based options presented in the modules which are sometimes in opposition to German guidelines [6].

Having no prior experience with a decision aid was not an implementation barrier in our study. In their updated systematic review, Légaré et al. found time constraints, patient characteristics, and the clinical situation to be the most often reported barriers for the implementation of shared decision making [37,38]. The most often reported facilitators were provider motivation, positive impact on the clinical process, and patient outcomes. Studies do not show that shared decision making necessarily requires more time [35,39,40]. We were also able to show that the length of most of the consultations was acceptable. A suspected negative impact on the doctor-patient relationship, a perceived disregard of professional status, and a possible threat to professional autonomy are implementation barriers discussed by Kaplan [17]. The results of the qualitative study of Watson et al. reveal that in order to implement decision aids in primary care a challenge might be the reconfiguration of the physician´s role in the physician-patient relationship. The reordering of power within this relationship might require more support than just training in implementation strategies [41].

Concerns about the comprehensiveness and up-to-dateness of decision aids might be another potential barrier for implementation [1]. There was a close cooperation between developers of patient decision aids and medical experts in the process of designing arriba-lib. Ease of use and a balanced presentation of evidence-based information emphasizing the freedom to choose might have resulted in our observation that prior experience with decision aids was not a critical variable in the implementation of an electronic library of decision aids. These conclusions are supported by a qualitative study on a computer delivered, theory based intervention for guideline implementation in general practice, although this is not a main goal of decision aids based on SDM. McDermott et al. found that the emerging reminders regarding guideline adherence were more likely to be accepted when physicians considered them to offer support and choice [42]. Physicians said that information should be presented in a condensed way and in an easy-to-understand format. The information should be tailored to the individual patient and physicians demanded to be able to choose among the presented information. Information on clinical topics, although evidence-based, must be offered in a way that patients and physicians maintain the impression of having the freedom to choose. Nevertheless, some physicians in our study raised concerns against some modules, e.g. atrial fibrillation, in which they saw a discrepancy between the evidence base and guidelines. This obviously resulted in a reluctance to use them.

Colombet et al. conducted a focus group study among general practitioners on an electronic decision aid that, for example, provided personalised risk estimates on cardiovascular prevention and diagnosis of depression [43]. Mentioned topics were the handling of the programme, the understanding of contents, and the acceptance of advice provided by the programme. It was shown that the understanding of risk information was highly variable in physicians. The authors advocate for training on the contents of the programme before feasibility testing, which we did in our study. Furthermore, the acceptance of evidence-based information for use in the decision making process should also be considered.

Evidence-based decision aids may offer support for physicians in the management of self-acquired information in patients. Baumgart describes a high ambivalence of physicians regarding the informed patient in her qualitative study [44]. Physicians report incorrect interpretations of information acquired by patients that often need a time consuming correction. The more complex the disease and the available treatment options, the more physicians appreciate information-seeking initiatives from their patients. Some physicians see a positive challenge in interacting with informed patients who might receive a greater sense of control in dealing with their disease. Modern information-oriented societies require a change from a paternalistic physician to an expert who accompanies patients in their search for and analysis of medical information. Evidence-based decision aids can play an important role in this process.

## Conclusions

Our study showed that it might be feasible to apply our electronic library of decision aids (arriba-lib) in the primary care context. The majority of physicians stated that the consultation length was not or acceptably extended.

A very detailed discussion of therapeutic options might be used in situations of high uncertainty or discordance in patients or physicians and therefore more often results in indecision.

Time constraints and having no prior experience with decision aids were not critical factors for implementation in our study. In this respect, ease of use and a balanced presentation of evidence-based information emphasizing the freedom to choose might be important. Evidence-based decision aids offer support for physicians in the management of medical information. Future studies should monitor the long-term adoption of arriba-lib in primary care physicians.

## Competing interests

The authors declare that they have no competing interests.

## Authors’ contributions

OH participated in the study design and coordination, developed the concept for data analysis, carried out the study, performed the statistical analyses, and drafted the manuscript. HK participated in the study design and coordination, the rationale for the data analyses, carried out the study, and helped to draft the manuscript. TK participated in the study design and coordination, the rationale for the data analyses, and helped to draft the manuscript. NDB participated in the study design and coordination, the rationale for the data analyses, and helped to draft the manuscript. All authors read and approved the final manuscript.

## Pre-publication history

The pre-publication history for this paper can be accessed here:

http://www.biomedcentral.com/1472-6947/12/48/prepub
